# Ceftaroline-Induced Thrombocytopenia: A Case Report

**DOI:** 10.7759/cureus.65488

**Published:** 2024-07-27

**Authors:** Diego Ortiz-Mendiguren, Ian Crespo-Orta, Mark Miranda-Márquez, Nicole Rivera-Bobe, Glorivette San Vicente-Morales

**Affiliations:** 1 Internal Medicine, Veterans Affairs Caribbean Healthcare System, San Juan, PRI; 2 Hematology/Oncology, Veterans Affairs Caribbean Healthcare System, San Juan, PRI; 3 Allergy and Immunology, Veterans Affairs Caribbean Healthcare System, San Juan, PRI

**Keywords:** drug-induced immune thrombocytopenia, abrupt, antibiotic, immune, induced, drug, severe, thrombocytopenia, ceftaroline

## Abstract

Drug-induced thrombocytopenia is a rare but significant adverse effect of certain medications, with the potential for severe bleeding, thrombosis, and death. This report discusses a rare case of severe thrombocytopenia induced by ceftaroline in a 69-year-old male with a history of atrial fibrillation on rivaroxaban and allergies to amoxicillin and sulfa drugs. Following the initiation of ceftaroline for left lower extremity purulent cellulitis, his platelet count dropped from 204,000 to 4,000 x 10³/μL within a day. Given the low platelet levels, anticoagulation therapy, and bleeding risk, immediate interventions and prompt recognition prevented major complications, highlighting the importance of recognizing drug-induced thrombocytopenia in clinical practice.

## Introduction

Drug-induced thrombocytopenia (DIT) is a rare but significant adverse effect of certain medications, with an incidence of approximately 10 cases per 1,000,000 people per year and a prevalence of about 25% in critically ill patients. Thrombocytopenia, characterized by a decreased number of platelets, can lead to severe bleeding or thrombosis. DIT often presents as an acute and severe decrease in platelet count, typically below 50 x 10³/μL, increasing the risk of spontaneous hemorrhages [1].

Common drugs causing DIT include beta-lactam antibiotics (e.g., penicillin, cephalosporins), NSAIDs, antiviral agents, chemotherapeutic agents, and antiplatelet drugs. The mechanisms of DIT include reduced platelet production due to bone marrow suppression and accelerated platelet destruction, which can be immune-mediated or non-immune, typically presenting five to 10 days post-exposure, with platelet counts often falling below 20,000 x 10³/μL [2].

Clinical symptoms range from asymptomatic to life-threatening hemorrhages. It is crucial to differentiate DIT from other causes like heparin-induced thrombocytopenia (HIT), ITP, and thrombotic thrombocytopenic purpura (TTP), as management strategies vary. Management involves prompt recognition, discontinuation of the offending drug, supportive care, and sometimes steroids or intravenous immunoglobin (IVIG). Diagnostic workup includes CBC, blood smear analysis, and tests for antiplatelet antibodies [1-2]. This report discusses a rare case of severe thrombocytopenia induced by ceftaroline in a 69-year-old male.

## Case presentation

A 69-year-old male with atrial fibrillation on rivaroxaban and a history of allergies to amoxicillin and sulfa drugs presented with partially treated left lower extremity purulent cellulitis. Given purulence and previous antibiotic treatment, ceftaroline was chosen. He was admitted to the ward and started on ceftaroline. A routine CBC revealed a platelet count drop from 204,000 to 30,000 x 10³/μL the following day. Repeat lab work during the day showed continued down-trending to 7,000 x 10³/μL. Given the concern of platelet clumping, a repeat laboratory on citrate was performed, which revealed platelets at 4,000 x 10³/μL.

Vital signs were notable for a heart rate of 74 beats per minute, blood pressure of 110/65 mmHg, and respiratory rate of 18 breaths per minute. The physical exam showed no petechiae or signs of bleeding. Labs revealed reticulocytes at 2.3%, elevated haptoglobin at 293 mg/dL, bilirubin within normal limits, elevated fibrinogen at 738 mg/dL, and mildly increased lactate dehydrogenase (LDH) at 239 u/L. The coagulation profile showed international normalized ratio (INR) prolongation at 1.74 (on rivaroxaban), and the peripheral smear was without schistocytes.

Given the severe thrombocytopenia, the primary care team immediately notified the hematology-oncology service. A PLASMIC score of 4 indicated a low probability of TTP, and there was no recent exposure to heparin products. Given the acute presentation, IV steroids with dexamethasone, removal of the probable offending agent, and a platelet transfusion were recommended. Ceftaroline was discontinued as it was the only new medication the patient had recently started, raising concern for medication-induced thrombocytopenia. Due to the decreasing platelet trend, anticoagulation therapy, and very high risk of bleeding, the patient was transferred to the medical intensive care unit for a higher level of care.

**Figure 1:**
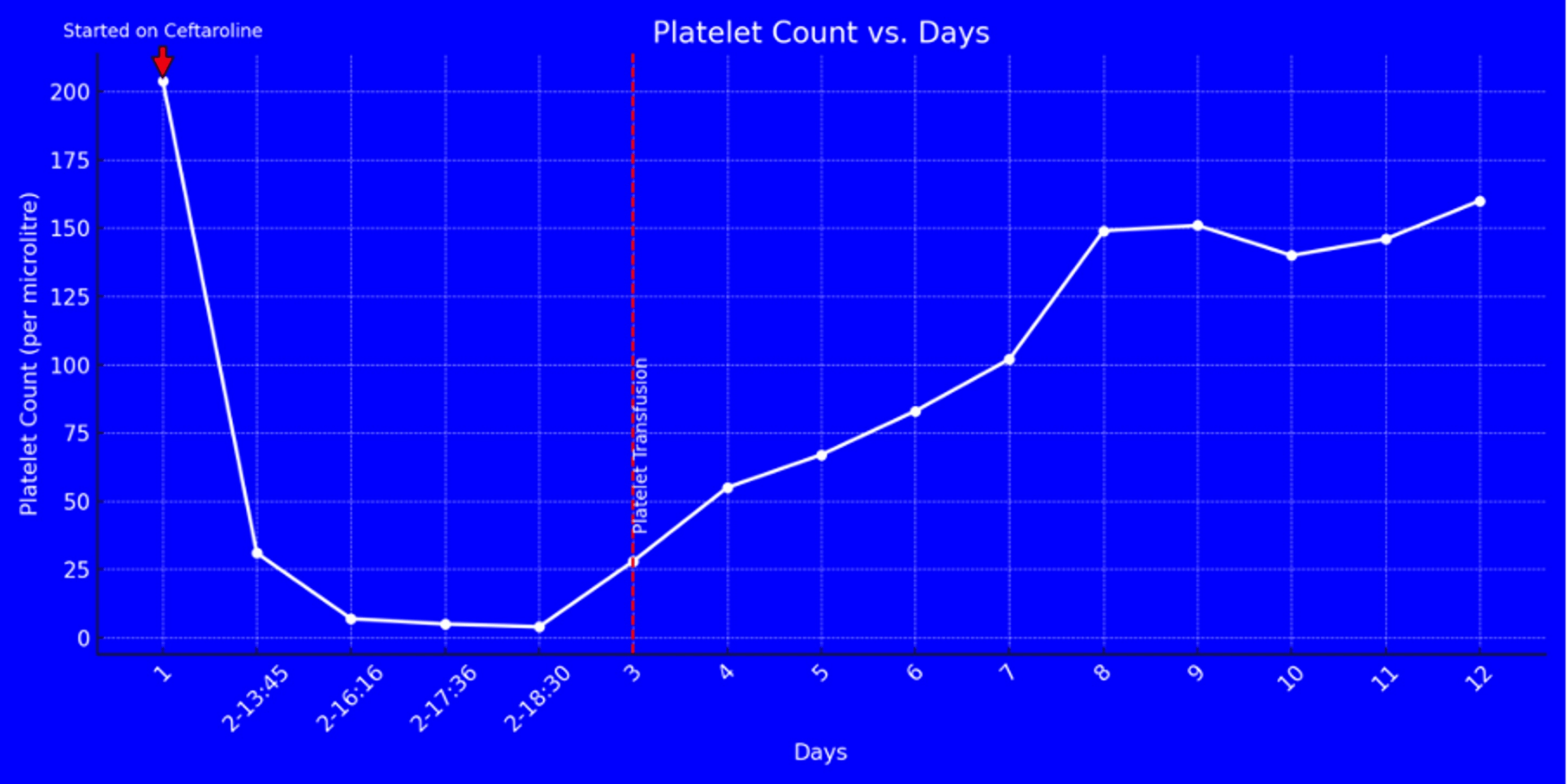
Platelet count and days: Following the initiation of ceftaroline, the platelet count steeply declined from the patient's baseline levels, reaching a minimum of 4 x 10³/μL. The platelet count then gradually increased, demonstrating recovery after the discontinuation of ceftaroline, platelet transfusion, and initiation of steroids.

Following the discontinuation of ceftaroline, the infectious disease service recommended transitioning the patient to ciprofloxacin and vancomycin. After stopping ceftaroline and changing the antibiotics, the patient's platelet levels gradually increased over the next few days. By day 8, the platelet count had risen to 150 x 10³/μL and continued to improve over time (Figure 1). The Naranjo adverse drug reaction scale scored ceftaroline at 7, suggesting it was the probable cause of the thrombocytopenia.

## Discussion

In the hospital setting, acutely ill patients are known to develop thrombocytopenia, defined as an acute drop of platelets below 150 x 10³/μL [3]. Thrombocytopenia is often asymptomatic but can present with bleeding complications such as petechiae, epistaxis, easy bruising, purpura, bleeding gums, and even life-threatening hemorrhage [4]. Consequently, identifying the cause of a rapidly dropping platelet count is crucial, as it can significantly impact patient outcomes.

**Figure 2:**
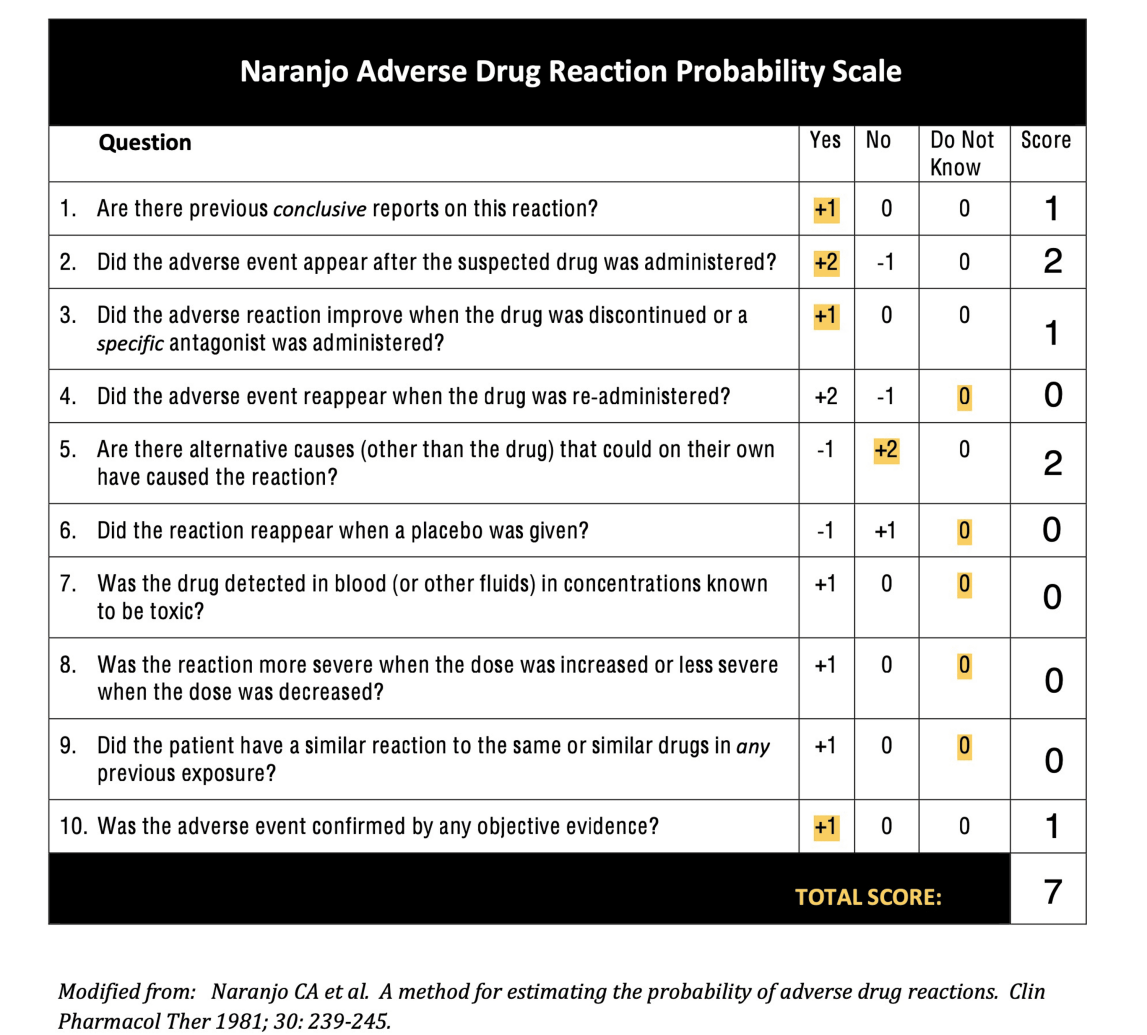
Naranjo Adverse Drug Reaction Probability Scale Modified from [5]

Ceftaroline-induced thrombocytopenia is a rare side effect, with only a handful of documented cases [4]. It is essential to consider this possibility in patients experiencing a sudden platelet drop shortly after starting the medication. Differentiating between Heparin-induced thrombocytopenia (HIT), Idiopathic Thrombocytopenic Purpura (ITP) is essential, and Thrombotic Thrombocytopenic Purpura (TTP), as their management strategies differ significantly. Drug-induced thrombocytopenia often results in more severe platelet count reductions compared to other causes, with levels below 20,000 x 10³/μL associated with a higher risk of bleeding. This rapid decline, with platelets dropping from 200,000 to 30,000 x 10³/μL in just one day, is consistent with what we observed in our patient. On the Naranjo scale (Figure 2), a score of 7 suggests ceftaroline as the probable cause of thrombocytopenia [5].

The exact mechanisms by which ceftaroline-induced thrombocytopenia is caused are not fully understood, but multiple possibilities exist. Beta-lactam antibiotics, particularly first to third-generation cephalosporins, can cause a form of thrombocytopenia through an immune-mediated reaction [4,6-8]. The beta-lactam ring in these antibiotics can bind to free haptens in the bloodstream, forming hapten-antibody complexes that act as antigens, triggering antibody production [4]. These antibodies are then thought to target and destroy platelets. Another possible explanation is that ceftaroline might also cause decreased platelet production through myelosuppression, although this requires further investigation [9-10].

Prompt recognition and management are essential to prevent serious complications. Including prompt cessation of the offending drug (ceftaroline in this case) and initiating appropriate treatment based on the underlying cause of the thrombocytopenia.

## Conclusions

This case highlights the critical need for awareness of drug-induced thrombocytopenia. Early identification and treatment can significantly improve patient outcomes, particularly in those on anticoagulants with an already increased bleeding risk.
